# Biology-Informed Recurrent Neural Network for Pandemic Prediction Using Multimodal Data

**DOI:** 10.3390/biomimetics8020158

**Published:** 2023-04-14

**Authors:** Zhiwei Ding, Feng Sha, Yi Zhang, Zhouwang Yang

**Affiliations:** 1 University of Science and Technology of China, Hefei 230022, China; zwding@mail.ustc.edu.cn; 2 Shenzhen Institute of Advanced Technology, Chinese Academy of Sciences, Shenzhen 518000, China; feng.sha@siat.ac.cn; 3 National Engineering Laboratory for Big Data Analysis and Applications, Peking University, Beijing 100091, China; zhangyi03@pku.edu.cn

**Keywords:** bio-inspired, back-projection, neural network, LSTM, deep learning, COVID-19, pandemic prediction

## Abstract

In the biomedical field, the time interval from infection to medical diagnosis is a random variable that obeys the log-normal distribution in general. Inspired by this biological law, we propose a novel back-projection infected–susceptible–infected-based long short-term memory (BPISI-LSTM) neural network for pandemic prediction. The multimodal data, including disease-related data and migration information, are used to model the impact of social contact on disease transmission. The proposed model not only predicts the number of confirmed cases, but also estimates the number of infected cases. We evaluate the proposed model on the COVID-19 datasets from India, Austria, and Indonesia. In terms of predicting the number of confirmed cases, our model outperforms the latest epidemiological modeling methods, such as vSIR, and intelligent algorithms, such as LSTM, for both short-term and long-term predictions, which shows the superiority of bio-inspired intelligent algorithms. In general, the use of mobility information improves the prediction accuracy of the model. Moreover, the number of infected cases in these three countries is also estimated, which is an unobservable but crucial indicator for the control of the pandemic.

## 1. Introduction

With the explosion of data available, obtaining the optimal solutions to data-driven problems is increasingly becoming a challenge. It has been recognized that the application of bio-inspired intelligent algorithms is necessary for addressing highly complex problems [1]. Until now, there are numerous algorithms inspired by nature or biological phenomena, such as neural networks, genetic algorithm (GE), ant colony algorithm (ACO), particle swarm optimization, and so on. They have various applications in solving engineering and biomedical problems [2,3]. Neural networks are usually defined as adaptive nonlinear data processing algorithms that combine multiple processing units connected within the network. The neural networks attempt to replicate the mechanism via which neurons are coded in intelligent organisms, such as human neurons. The long short-term memory (LSTM) model is one of the popular neural networks [4,5].

The prevention and control of infectious diseases is an important research topic in biomedicine. In recent years, infectious diseases have occurred from time to time, such as influenza A (H1N1), the coronavirus disease 2019 (COVID-19), and so on. The outbreak of COVID-19 has spread across the world. Many countries adopted various forms of lockdown to reduce social contact and thus inhibit the spread of coronavirus; this disrupted supply chains, depressed consumer demand, and put millions out of work [6,7]. Moreover, the spread of influenza has continued to show an upward trend in multiple provinces across China, with outbreaks of the influenza A (H1N1) virus being reported at many schools in several areas in February 2023 [8]. Thus, it is important to model infectious diseases to predict their trends. On the one hand, the outbreak of infectious diseases harms people’s health, and predicting the number of confirmed cases in advance can provide decision making support for prevention and control. On the other hand, estimating the number of existing infected cases could help allocate medical resources, such as beds and ventilators.

New problems emerge one after another, and traditional algorithms often cannot solve them effectively. By analyzing the problem, we can design personalized algorithms. In the field of infectious diseases, the time interval from infection to medical diagnosis is a random variable that obeys the specific log-normal distribution confirmed by previous research [9]. Inspired by this biological law, the back-projection algorithm is proposed to estimate the number of infected cases. Analyzing the development law and predicting the pandemic provides useful insights to policymakers, and allows them to make informed decisions on allocating limited resources, controlling outbreaks, and ensuring the safety of the general public. Various population information and social factors, such as community mobility, population density, the awareness of wearing masks, and so on, have an impact on the spread of infectious diseases. We intuit that the use of multisource data will provide a highly meaningful avenue for modeling and forecasting.

In this paper, we first formulate a modified back-projection model inspired by the law of infectious disease transmission, and then propose a hybrid bio-inspired architecture combining modified back-projection and the recurrent neural network for pandemic prediction. The main contributions of this paper are summarized as follows:

- We propose a novel hybrid bio-inspired neural network model that not only predicts the number of new daily confirmed cases, but also estimates the number of new daily infected cases.
- Using the multimodal data, we design the LSTM module to estimate the time-varying infection rates in the infected–susceptible–infected (ISI) module. This is more practical and flexible compared with the common curve fitting methods.
- The proposed model, BPISI-LSTM, outperforms the popular epidemic prediction models on real-word datasets with different sizes of prediction window.

The remainder of this paper is organized as follows. Section 2 outlines the related work in pandemic prediction, especially for COVID-19. Section 3 describes the framework of the proposed model and details its mathematical theory. Section 4 provides the experimental results of predicting confirmed cases using the multimodal data of three countries. Section 5 discusses the superiority of the model in estimating the number of infected cases. The conclusion is provided in Section 6.

## 2. Related Work

We focus on the related methods of infectious disease prediction, which are mainly divided into compartmental mathematical models, mechanistic statistical models, and deep learning models.

Compartmental mathematical models include the susceptible–infected–recovered (SIR) model and its derived models, such as the susceptible–exposed–infected–recovered (SEIR) model. These models divide the population into exclusive groups and define the progress among the different groups through ordinary differential equations. Kim et al. [10] developed a novel SEIR model based on the Coxian distribution approximating the distribution of the incubation. The model is adaptive in order to resolve the various realistic epidemic predictions, since all types of incubation periods are approximated by the Coxian distribution. However, several parameters need to be fitted using real epidemic data, which is a non-trivial problem. Sun et al. [11] proposed a more generalized version of the SIR model, where the infection rate and the recovery rate both vary with time. The reciprocal regression is used to estimate the infection rate, and the recovery rate curve is fitted using the last five data points. The model is evaluated to track the epidemic of COVID-19 in 30 provinces in China and 15 cities in Hubei province. Chen et al. [12] also derived a time-dependent SIR model that tracks the transmission and recovery rate at time *t*. Due to the existence of asymptomatic infections of COVID-19, they extend the model by considering two types of infected persons: detectable and undetectable infected persons. Giordano et al. [13] proposed the compartmental model considering eight stages of infection. The model discriminates the infected individuals according to whether they have been diagnosed and the severity of their symptoms. The prediction of the model, in the long run, is not very sensitive to the initial conditions, but it is sensitive to parameters in the model estimated using empirical data.

Back-projection is representative of mechanistic statistical models that were developed to estimate the human immunodeficiency virus (HIV) incidence using surveillance data on acquired immunodeficiency syndrome (AIDS) diagnosis [9]. Becker et al. [14] modified classical back-projection using the multiplicative method to model the age-specific relative risk of HIV infection. The smoothed expectation maximization (EM) algorithm is applied to solve the modified back-projection model. Chau et al. [15] proposed modified back-projection based only on the number of HIV diagnoses. The model rectifies some of the shortcomings of the original back-projection method based on AIDS data alone. McEwan et al. [16] applied the classical back-projection approach to estimate the number of patients living with chronic hepatitis C virus (HCV) infection in Taiwan. Moreover, they quantified the expected numbers in each of the five METAVIR fibrosis stages. Back-projection was also used to analyze the surveillance data of COVID-19 diagnoses for different regions, such as Hong Kong [17], Australia [18], and so on. However, it is difficult to estimate the recent infection cases precisely using the classical back-projection model, let alone predict the number of new daily infected cases in the future. There are two unavoidable sources of uncertainty. First, the prediction involves unknown future infection rates. Second, little is known about the recent infection rate, which is the consequence of the long and variable incubation period of the infectious disease, and cannot be overcome by statistical ingenuity [14].

Neural network methods, such as long short-term memory (LSTM) [19], the graph neural network [20,21,22], and so on, have been extensively used to predict pandemics in recent years. To predict the influenza-like illness (ILI) in Guangzhou, Fu et al. [23] designed a multi-channel LSTM network to extract fused descriptors from multiple types of inputs. They further improved the prediction accuracy by adding an attention mechanism, allowing the model to handle the relationship of multiple input streams more appropriately. Deng et al. [24] designed a message-passing framework to combine learned feature embeddings and an attention matrix to model disease propagation over time. They evaluated the model on real epidemiological data and validated its effectiveness. However, the proposed model only uses flu disease data and geographic location data, thus ignoring external features such as weather, social factors, and population migration. Tian et al. [25] proposed the COVID-Net network, combining both LSTM cells and gated recurrent unit (GRU) cells, which takes the five risk factors and disease-related history data as the input. Wu et al. [26] developed a deep learning framework combining the recurrent neural network (RNN), the convolutional neural network (CNN), and residual links for epidemiological predictions. In the proposed framework, RNN captures the long-term correlation and CNN fuses information from different sources. The residual structure is applied to prevent overfitting issues. Their approach shows excellent performance on real epidemic data. These pure deep learning models are data-driven without any epidemic mechanism. They are likely to predict the short-term trend of the epidemic, while have poor long-term prediction precision.

In this paper, we aim to overcome these limitations by combining the mechanistic model and the deep learning model. Different approaches following this idea have been proposed for several applications; for example, Zheng et al. [27] proposed a hybrid artificial intelligence (AI) model, including a susceptible–infected module, LSTM module, and natural language processing (NLP) module, for COVID-19 prediction. In addition to infectious disease data, the hybrid model takes the prevention and control measures and related news reports as input, considering the effects of prevention and control measures. Gatta et al. [28] proposed a novel machine-learning-based framework able to estimate the parameters of compartmental models, such as contact rates and recovery rates, based on static and dynamic features of places. However, these methods cannot estimate the number of infected cases. In this paper, the law of infectious disease transmission and the deep learning model are combined to predict the numbers of confirmed and infected cases.

## 3. Methodology

In this section, the proposed methodology for designing the hybrid model for COVID-19 pandemic prediction is presented.

### 3.1. Framework of the Hybrid Model

The compartmental models based on differential equations divide the population into exclusive groups, define the transition from one group to another, and predict the epidemic. One of the most extensively used compartmental models is the susceptible–exposed–infected–recovered (SEIR) model, which does not distinguish the confirmed cases and infected cases. In practice, the model is solved using the confirmed cases rather than the infected cases due to the unobservability of the infected cases. Therefore, the number of infected cases obtained by the SEIR model is actually the number of confirmed cases. However, the estimation of infected cases is a crucial indicator in terms of informing policymakers and thus controlling the epidemic. Based on the retrospective method, the back-projection models the transition from the infected cases to the confirmed ones, and estimates the number of new daily infected cases. Thus, we take back-projection as the basic module of the proposed hybrid model.

Unfortunately, the weakness of back-projection also exists in the retrospective method, that is, the estimators of infected cases from day $t-t_{0}$ to *t* are inaccurate under the assumption that *t* is the latest day, where $t_{0}$ is a constant related to the transmission capacity of the coronavirus. Due to the time lag from infection to diagnosis, the estimation of the infected cases from day $t-t_{0}$ to *t* involves the information of confirmed cases in the future. The naive back-projection cannot deal with this problem.

In addition to the conversion from infection to diagnosis, the development law also exists within the infection cases. Under the prevention and control measures, the newly infected cases at the current moment are infected by the newly infected cases in recent days. Under this assumption, the ISI model is proposed to calculate the infection rate to revise the inaccurate estimation of new daily infected cases from day $t-t_{0}$ to *t*. The basic principle of the ISI model is to use the ratio of the number of newly infected cases at day *t* to the cumulative number of new confirmed cases over different time scales before day *t* to calculate the infection rate and establish an epidemic model.

The infection rate of coronavirus varies with time. Limited by the ability of fitting data of common functions, such as exponential functions, power functions, and so on, we use the LSTM model to predict infection rates from day $t-t_{0}$ to *t*. To include the impact of mobility on the spread of the pandemic, community mobility data collected via Google are used as additional features as the input of our LSTM module, in addition to disease-related historical information.

**Figure 1:**
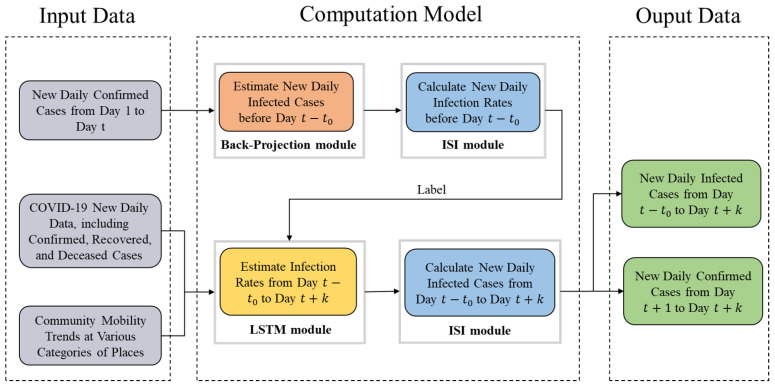
Hybrid bio-inspired model for pandemic prediction using multimodal data. ISI stands for infected–susceptible–infected. LSTM stands for long short-term memory.

The output of the LSTM model, i.e., the infection rates from day $t-t_{0}$ to t+k, is used in the ISI model to estimate the infected cases from day $t-t_{0}$ to t+k, and then the confirmed cases are also calculated. The proposed framework is shown in Figure 1.

### 3.2. Back-Projection Module

Individuals infected with coronavirus will be clinically diagnosed several days later, either because they feel unwell and actively undergo testing with a nucleic acid reagent, or because the government implements a national screening policy and they are passively diagnosed. In short, by collecting the nucleic acid test data from medical institutions, the new daily confirmed cases can be calculated, while the new daily infected cases are unobserved. Back-projection based on a retrospective approach estimates the new daily infected cases up to the present, forming the basis for prediction of the infected cases. The basic principle of the retrospective approach is that the new daily diagnosed individuals come from the new daily infected individuals from previous days with a certain probability.

Let I(t) denote the unobserved number of individuals infected with coronavirus on day *t*. The number of COVID-19 cases diagnosed on the day *t* is denoted by C(t). The method of back-projection is based on the following assumptions.

1. Infected individuals must be confirmed later, that is, death before diagnosis is not considered.
2. The ${\left\{I\left(t\right)\right\}}_{t=1}^{T}$ outputs are assumed to be independent Poisson variables.
3. The time from infection to diagnosis, denoted by *X*, is a log-normal random variable, which is the same irrespective of when the individual is infected.
  $$X∼ln(N\left(\mu ,\sigma^{2}\right))$$
  where μ=7.2 and $\sigma =\left(ln\left(15.2\right)-ln\left(7.2\right)\right)/\left(Z_{0.95}\right)$, and $Z_{0.95}$ is the 0.95 upper quantile of the standard normal distribution.

Under Assumption 3, we have

(1) P(confirmedatt|infectedats)=f(t−s|s)=f(t−s)

where f(·) is the discretized log-normal density function.

Based on the above assumptions, we have

(2) $$E\left[C\left(t\right)|I\left(1\right),I\left(2\right),\ldots ,I\left(t\right)\right]=\sum_{s=1}^{t}I\left(s\right)f\left(t-s;\mu ,\sigma \right)$$

Then

(3) $$\begin{array}{cc} E[C(t)] & =E[E[C(t)|I(1),I(2),\ldots ,I(t)]]\\ \; & =\sum_{s=1}^{t}E\left[I\left(s\right)\right]f\left(t-s;\mu ,\sigma \right) \end{array}$$

Thus, the mean number of confirmed cases on day *t* is

(4) $$d_{t}=\sum_{s=1}^{t}i_{s}f\left(t-s;\mu ,\sigma \right)$$

where $d_{t}=E\left[C\left(t\right)\right]$ and $i_{t}=E\left[I\left(t\right)\right]$.

Assumption 2 implies that C(1),C(2),…,C(t) are also independent Poisson variables. Corresponding to the observed daily confirmed cases, $c_{1},c_{2},\ldots ,c_{t}$, we then have the likelihood function

(5) $$\begin{array}{cc} \; & L\{C\left(1\right),C\left(2\right),\ldots ,C\left(t\right);i_{1},i_{2},\ldots ,i_{t}\}\\ \; & =P(C\left(1\right)=c_{1},C\left(2\right)=c_{2},\ldots ,C\left(t\right)=c_{t})\\ \; & =P\left(C\left(1\right)=c_{1}\right)P\left(C\left(2\right)=c_{2}\right)\ldots P\left(C\left(t\right)=c_{t}\right)\\ \; & \propto \prod_{s=1}^{t}\left\{d_{s}^{c_{s}}\cdot exp\left(-d_{s}\right)\right\}\\ \; & \propto \prod_{s=1}^{t}\left\{{\left[\sum_{r=1}^{s}i_{r}f\left(t-r\right)\right]}^{c_{s}}\cdot exp\left(-\sum_{r=1}^{s}i_{r}f\left(t-r\right)\right)\right\} \end{array}$$

Maximization of the likelihood function for the $i_{r}$ via the EM algorithm always leads to non-negative estimates. However, there is a problem of large fluctuations within the sequence ${\left\{i_{r}\right\}}_{r=1}^{t}$ using a naive EM algorithm, so we introduce smoothing in each iteration [9]. The specific steps are as follows. Let *T* represent today’s date.

Expectation Step: The posterior expectation of the number of patients who are infected on day *t* and confirmed on day t+q is calculated as follows.

(6) $$E\left[N_{t,q}|c_{1},c_{2},\ldots ,c_{T}\right]=c_{t+q}\frac{i_{r}f\left(q\right)}{\sum_{s=1}^{t+q}i_{s}f\left(t+q-s\right)}$$

Maximum Step:

(7) $$i_{t}^{\left[k+1\right]}=\frac{{\tilde{i}}_{t}^{\left[k\right]}}{\sum_{q=0}^{T-t}f\left(q\right)}\sum_{q=0}^{T-t}\frac{c_{t+q}f\left(q\right)}{\sum_{s=1}^{t+q}{\tilde{i}}_{s}^{\left[k\right]}f\left(t+q-s\right)}$$

where ${\tilde{i_{t}}}^{\left[k\right]}$ is the smoothed estimator of the *k*th iteration.

Smooth Step:

(8) $${\tilde{i}}_{t}^{\left[k+1\right]}=\sum_{s=0}^{r}w_{s}i_{t+s-\frac{r}{2}}^{\left[k+1\right]}$$

where $w_{s}$ is the symmetric binomial weight, that is, $w_{s}=C_{r}^{s}/2^{r},s=0,1,\ldots ,r$.

When *t* is close to 1 or *T*, the subscript of $i_{t+s-\frac{r}{2}}^{\left[k+1\right]}$ may be out of range. To avoid this situation, we make the provision for the potential subscript out of range: $i_{t+s-\frac{r}{2}}^{\left[k+1\right]}=0$ when $t+s-\frac{r}{2}<1$, and $i_{t+s-\frac{r}{2^{\left[k+1\right]}}}=i_{T}^{\left[k+1\right]}$ when $t+s-\frac{r}{2}>T$.

Stopping Criterion: given a constant $T_{0},T_{0}\leq T$ and the upper bound of the accepted error $\epsilon_{0}$, the algorithm fails if $\sum_{t=1}^{T_{0}}\frac{|i_{t}^{\left[k+1\right]}-i_{t}^{\left[k\right]}|}{i_{t}^{\left[k\right]}}<\epsilon_{0}$.

Here we take ϵ=0.005, the size of smoothing window r=2, and $T_{0}=T-1$. The likelihood function in this paper is a concave function, and the smoothing function in the Smooth Step is a linear function; thus, the EM algorithm converges and the final convergent point is unique. The proof is omitted here, please see the References section for details.

According to ${\tilde{d}}_{t}=\sum_{s=1}^{t}{\tilde{i}}_{s}f\left(t-s\right)$, we can calculate the estimated number of new daily confirmed cases ${\tilde{d}}_{s},t=1,2,\ldots ,T$ after obtaining ${\tilde{i}}_{s}$.

### 3.3. Infected–Susceptible–Infected Module

Individuals infected with the coronavirus will spread the virus to those who are susceptible through social contact. Since the infected individuals will show abnormal symptoms, such as fever, dry cough, fatigue, etc., they will eventually accept the nucleic acid reagent test and be diagnosed.

The observation period of COVID-19 is 14 days, so we assume that the maximum length of time for an infected individual from being infected to no longer spreading the virus is 14 days, that is, all new daily infected cases are infected by patients infected in the past 14 days.

Most people under epidemiological investigations will be quarantined, observed, and tested with a nucleic acid reagent. It takes at least two positive tests for a patient to be diagnosed as positive for COVID-19. Therefore, we speculate that most of the confirmed cases have been quarantined at least 3 days before being diagnosed, and are unable to infect others [27], which means that most of the infected persons were not infected by another infected individual who was infected 11 days previously. Therefore, for each day *t*, this paper examines the infection rate of new daily infected cases in the past 10 days relative to the infected cases of day *t*.

The infected–susceptible–infected (ISI) model is also based on the retrospective method, in which the newly infected cases on day *t* were infected by the newly infected cases on day t−1,t−2,…,t−10. Therefore, we can use the following formula to describe

(9) $$i_{t}=\beta \left(t;w\right)\sum_{s=1}^{10}\alpha_{s}i_{t-s}$$

where β(s;w) is the infection rate of day *s* and *w* is the parameter, and $\alpha_{s}$ is the weight assigned to different time points.

We calculate the infection rate according to Equation (9).

### 3.4. Long Short-Term Memory

**Figure 2:**
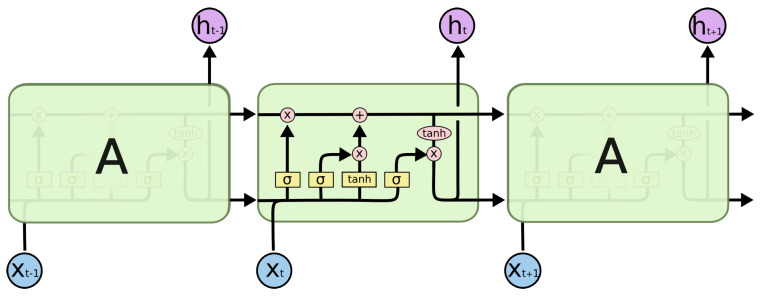
Detailed schematic of the LSTM block.

The recurrent neural network can dynamically incorporate experience due to internal recurrence. Unlike conventional RNN, LSTM can solve the problem of vanishing and exploding gradients. A LSTM memory cell has four units: input gate, output gate, forget gate, and a self-recurrent neuron. LSTM is implemented by following a composite function, and the detailed pipeline is shown in Figure 2.

(10) $$\left\{\begin{array}{c} i_{t}=\sigma \left(W_{ii}X_{t}+b_{ii}+W_{hi}h_{t-1}+b_{hi}\right)\\ f_{t}=\sigma \left(W_{if}X_{T}+b_{if}+W_{hf}h_{t-1}+b_{hf}\right)\\ g_{t}=tanh\left(W_{ig}X_{t}+b_{ig}+W_{hg}h_{t-1}+b_{hg}\right)\\ o_{t}=\sigma \left(W_{io}X_{t}+b_{io}+W_{ho}h_{t-1}+b_{ho}\right)\\ c_{t}=f_{t}⨀c_{t-1}+i_{t}⨀g_{t}\\ h_{t}=o_{t}⨀tanh\left(c_{t}\right) \end{array}\right.$$

where σ(·) represents the logistic sigmoid function; *i*, *o*, *f*, and *c* represent the input gate, forget gate, output gate, and cell input activation vectors, respectively; *h* represents the hidden vector. The weight matrix subscripts have an intuitive meaning; for example, $W_{hi}$ represents the hidden input gate matrix, etc.

## 4. Results

We evaluate the proposed model on multimodal data of developed and developing countries. Experiments applying the LSTM module of the BPISI-LSTM network were run on an NVIDIA GeForce RTX 3060 GPU with Pytorch 1.7.1. The Adam optimizer was adopted during the optimization. To evaluate the efficiency of the hybrid framework, we compared it against other popular models and conducted an ablation analysis. The code is publicly available on GitHub (https://github.com/ryannuan, accessed on 15 January 2023).

### 4.1. Data Description

We use the multimodal data, which are listed in Table 1, as the input of the models. The following two datasets were utilized to obtain disease-related and mobility features.

*COVID-19 Daily Dataset* (https://github.com/CSSEGISandData/COVID-19, accessed on 10 January 2023). This dataset was released by Johns Hopkins University and updated daily. By calculation, we obtained the disease-related features, including the new daily number of confirmed, dead, recovered, and hospitalized cases. The dataset reflects the development of the epidemic.

*Community Mobility Dataset* (https://www.google.com/covid19/mobility/, accessed on 10 January 2023). This dataset, released by Google, summarizes mobility trends at various categories of places that are aggregated at the country level. The categories of places include grocery stores and pharmacies, parks, transit stations, workplaces, residential areas, and retail and recreation areas. The dataset shows how visits and length of stay at different places change compared to a baseline. The baseline is the median value during the 5-week period from 3 January to 6 February 2020.

India and Indonesia were selected as representatives of developing countries, and Austria was selected as a representative of developed countries. We evaluated the proposed model using data from India, Austria, and Indonesia. For India, we utilized the dataset from 30 January to 22 November 2020. For Indonesia, we utilized the dataset from 2 March 2020 to 2 May 2021. For Indonesia, we utilized the dataset from 25 February 2020 to 2 May 2021.

### 4.2. Implementation Details

**Figure 3:**
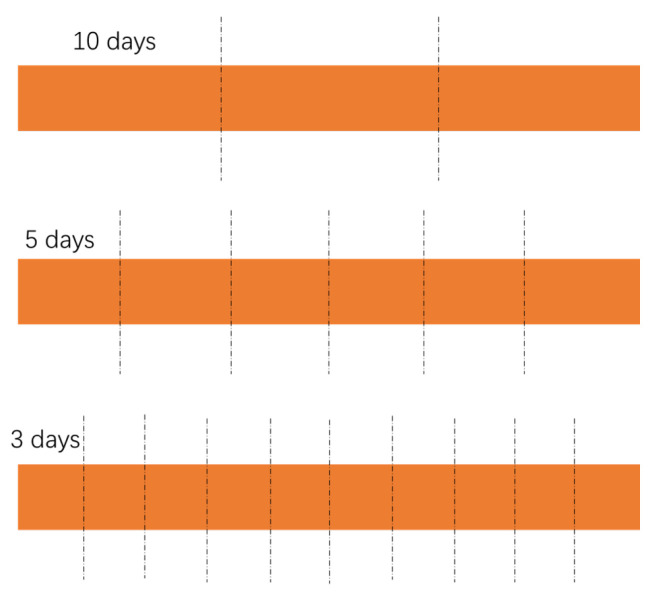
The splitting strategy of the testing set (the last 30 days).

*Data Preprocessing*. Data for the last 30 days were reserved as the testing set, and the remaining data were randomly divided into the training set and validation set according to the ratio of 9:1. To evaluate the model, we adopted three sizes of prediction window (3, 5, 10 days), and thus, the test set was split into 10, 6, 3 samples, correspondingly, as shown in Figure 3. For the inputs of the LSTM module, disease-related features and mobility information were all normalized to [0, 1].

*Evaluation Criteria*. Following the previous work [20], the root mean squared error (RMSE), mean absolute error (MAE), and mean absolute percentage error (MAPE) were adopted to measure the prediction performances. The details are as follows:

$$\mathrm{RMSE}=\sqrt{\frac{1}{n}\sum_{i=1}^{n}{\left({\hat{y}}_{i}-y_{i}\right)}^{2}}$$

$$\mathrm{MAE}=\frac{1}{n}\sum_{i=1}^{n}\left|{\hat{y}}_{i}-y_{i}\right|$$

$$\mathrm{MAPE}=\frac{1}{n}\sum_{i=1}^{n}\frac{|{\hat{y}}_{i}-y_{i}|}{y_{i}}$$

where ${\hat{y}}_{i}$ is the number of confirmed cases predicted by the model, and $y_{i}$ is the actual number of confirmed cases officially announced.

### 4.3. Prediction of the Confirmed Cases

To evaluate the benefits of the proposed hybrid model, we compared it against the following popular methods as baselines for predicting the number of confirmed cases. ’No Mob BPISI-LSTM’ indicates that the BPISI-LSTM network does not utilize the community mobility dataset. To rule out randomness, we ran models five times under the same hyper-parameter settings and report the average metrics for LSTM, No Mob BPISI-LSTM, and BPISI-LSTM.

*vSIR* [8]. vSIR is a varying coefficient susceptible–infected–removal model, where the infection rate and recovery rate both vary with time.

**Figure 4:**
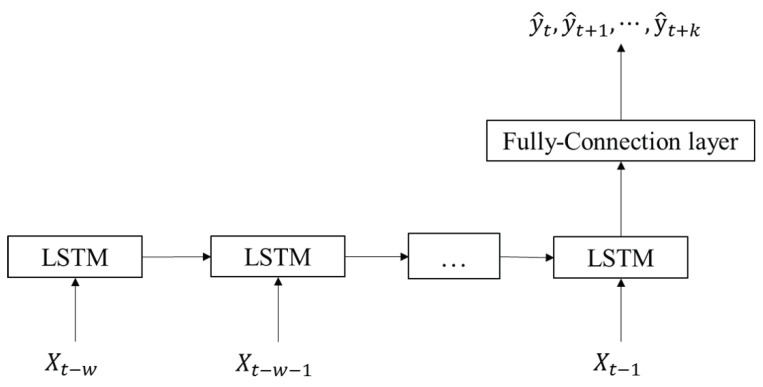
Structure of the LSTM baseline.

*LSTM*. Our LSTM baseline contains a stack of one LSTM layer with 16 units and a dense layer. The LSTM takes disease-related and mobility features in the past *w* days as the input. The dense layer takes the final output from the LSTM layer and outputs a vector with the size *k* (3, 5, or 10), which is the predicted number of confirmed cases in *k* days. The structure of the LSTM baseline is shown in Figure 4.

*BPISI*. The LSTM module in the BPISI-LSTM network is replaced by the two-parameter exponential function β(t;a,b) to fit the infection rate in Equation (9).

(11) β(t;w)=β(t;a,b)=a∗exp(−bt)

where a>0 and b>0.

The evaluation metrics of predicting the confirmed cases in India, Austria, and Indonesia are presented in Table 2, Table 3 and Table 4, respectively. Firstly, we found that the BPISI-LSTM network successfully outperforms vSIR and LSTM for both short-term and long-term predictions. Secondly, the prediction errors of vSIR and LSTM increase significantly with the prediction window, while the BPISI-LSTM remains at a low level. It is the hybrid framework of the BPISI-LSTM network, combining the development laws and powerful fitting ability of the LSTM module, that plays a huge role. Thirdly, due to data inaccuracy and information redundancy, additional mobility data may sometimes yield similar performance. Fourthly, the LSTM module can capture the dynamics of multisource features and improve the performance of the model significantly.

## 5. Discussion

Most epidemic models can only predict the number of confirmed cases based on historical disease-related data. However, our model predicts the numbers of both confirmed and infected cases. We used BPISI-LSTM to estimate the numbers of infected cases in India, Austria, and Indonesia, respectively. We plotted the numbers of confirmed and infected cases over time, with the red line representing the estimated infected cases and the green dashed line representing the real confirmed cases.

**Figure 5:**
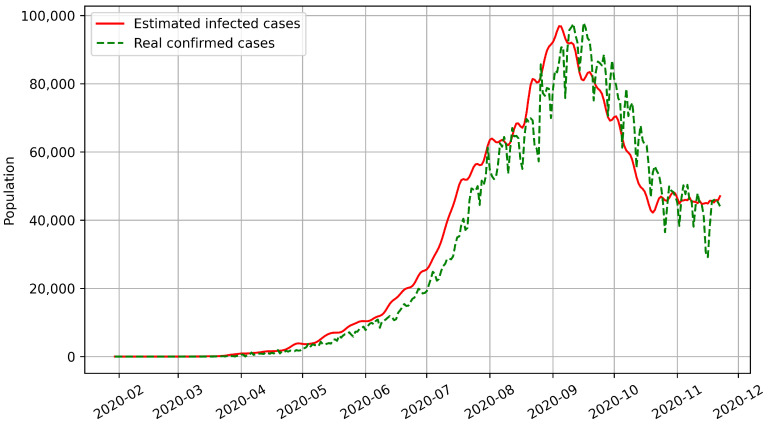
Estimated numbers of infected cases for India.

Firstly, the numbers of infected and confirmed cases in India from the onset of COVID-19 to 22 November 2020 are shown in Figure 5. As of 22 November 2020, the peak of infection occurred in India in early September 2020.

**Figure 6:**
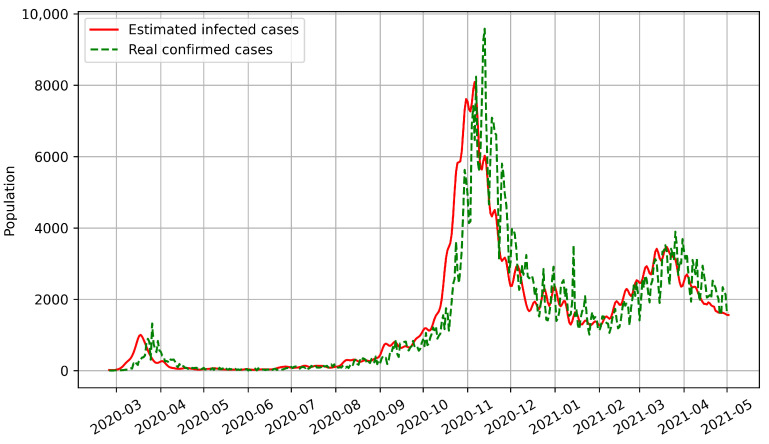
Estimated numbers of infected cases for Austria.

Secondly, the numbers of infected and confirmed cases in Austria between the onset of COVID-19 and 2 May 2021 are shown in Figure 6. Up to 2 May 2021, there have been three infection peaks in Austria, in mid-March 2020, early November 2020, and early March 2021, respectively.

**Figure 7:**
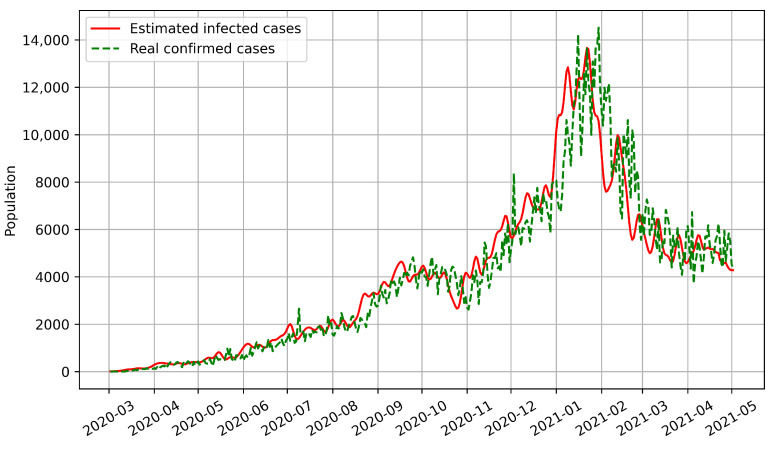
Estimated numbers of infected cases for Indonesia.

Thirdly, the numbers of infected and confirmed cases in Indonesia between the onset of COVID-19 and 2 May 2021 are shown in Figure 7. As of 2 May 2021, two peaks of infection occurred in Indonesia, in mid-September 2020 and mid-January 2021.

From the above figures, we can see that the curve of confirmed cases has an overall delay compared with the curve of infected cases, which indicates that the number of infected cases is a more sensitive indicator. Thus, the estimation of infected cases can inform us on how to prevent and control the pandemic in advance.

The time interval from infection to medical diagnosis is a random variable that obeys the log-normal distribution. Inspired by this biomedical law, our designed bio-inspired intelligent algorithms show the powerful ability to estimate the number of infected cases and predict the number of confirmed cases. Experimental results show that the prediction performance of intelligent algorithms can be further improved based on biological laws.

## 6. Conclusions

By analyzing the transmission mechanism of COVID-19, we used multimodal data to predict confirmed cases and infected cases. On the one hand, the time interval from infection to medical diagnosis is a random variable that obeys the specific log-normal distribution. On the other hand, in addition to the daily disease-related data, movement trends over time by geography also provide a new perspective for epidemic prediction. Based on these two motivations, we propose a back-projection-based bio-inspired hybrid model (BPISI-LSTM). The model takes disease-related data and social migration data as input, and these data are encoded by LSTM and concatenated to obtain the multimodal feature for prediction. We validate the effectiveness of the proposed model on multimodal datasets of developed and developing countries. Firstly, our experiment results show that the utilization of biological laws, LSTM modules, and multimodal data improves the prediction accuracy of the confirmed cases. Secondly, compared with other models that can only predict the number of confirmed cases, BPISI-LSTM also estimates the number of infections, and thus predicts the pandemic in advance.

Mobility and disease-related features are both used in the model. We encourage future researches that explore more external features, such as, the prevalence of wearing masks, changes in the weather, and so on. Moreover, this modeling framework can be readily extended. For example, the LSTM module can be replaced by the graph neural network, which may better capture the mobility information between regions and attributes of regions such as the population and medical resources.

## Figures and Tables

**Table 1:** Multimodal data description.

	Feature	Description	Note
Disease-related	Cumulative Confirmed	The cumulated number of confirmed cases	-
	Cumulative Recovered	The cumulated number of recovered cases	-
	Cumulative Dead	The cumulated number of dead cases	-
	Infected	The number of new daily infected cases	Unobservable
	Confirmed	The number of new daily confirmed cases	Input of the model
	Recovered	The number of new daily recovered cases	Input of the model
	Dead	The number of new daily dead cases	Input of the model
	Hospitalized	The number of COVID-19 patients at hospital	Input of the model
Mobility-related	Grocery and Pharmacy	Mobility trends at grocery and pharmacy	Input of the model
	Parks	Mobility trends at parks	Input of the model
	Transit Stations	Mobility trends at transit stations	Input of the model
	Workplaces	Mobility trends at workplaces	Input of the model
	Residential	Mobility trends at residential places	Input of the model
	Retail and Recreation	Mobility trends at retail and recreation	Input of the model

**Table 2:** Results of confirmed cases in India under different prediction windows.

Metrics	Model	3 Days	5 Days	10 Days
RMSE	vSIR	34,099	32,856	30,299
	LSTM	5897	6996	12,168
	BPISI	12,937	13,857	16,387
	No Mob BPISI-LSTM	5264	5906	6043
	BPISI-LSTM	4908	5364	5805
MAE	vSIR	33,900	32,597	29,342
	LSTM	5324	6172	11,229
	BPISI	12,415	13,329	16,387
	No Mob BPISI-LSTM	4696	5065	4780
	BPISI-LSTM	4120	4388	4477
MAPE	vSIR	0.7855	0.7600	0.6879
	LSTM	0.1286	0.1576	0.2016
	BPISI	0.2731	0.2955	0.3398
	No Mob BPISI-LSTM	0.1166	0.1241	0.1183
	BPISI-LSTM	0.1066	0.1115	0.1130

**Table 3:** Results of confirmed cases in Austria under different prediction windows.

Metrics	Model	3 Days	5 Days	10 Days
RMSE	vSIR	378	532	984
	LSTM	608	681	739
	BPISI	400	418	447
	No Mob BPISI-LSTM	334	349	352
	BPISI-LSTM	328	346	335
MAE	vSIR	333	440	858
	LSTM	526	604	625
	BPISI	331	356	373
	No Mob BPISI-LSTM	314	300	306
	BPISI-LSTM	295	297	300
MAPE	vSIR	0.1411	0.1865	0.3717
	LSTM	0.1775	0.2024	0.2040
	BPISI	0.1595	0.1698	0.1795
	No Mob BPISI-LSTM	0.1399	0.1306	0.1332
	BPISI-LSTM	0.1326	0.1294	0.1320

**Table 4:** Results of confirmed cases in Indonesia under different prediction windows.

Metrics	Model	3 Days	5 Days	10 Days
RMSE	vSIR	880	871	1175
	LSTM	810	842	889
	BPISI	1495	1598	1773
	No Mob BPISI-LSTM	843	837	917
	BPISI-LSTM	785	800	857
MAE	vSIR	774	736	1049
	LSTM	728	693	727
	BPISI	1357	1467	1638
	No Mob BPISI-LSTM	739	710	740
	BPISI-LSTM	695	668	699
MAPE	vSIR	0.1484	0.1372	0.1968
	LSTM	0.1286	0.1381	0.1503
	BPISI	0.2508	0.2738	0.3074
	No Mob BPISI-LSTM	0.1367	0.1249	0.1353
	BPISI-LSTM	0.1302	0.1248	0.1278

## Data Availability

Not applicable.

## References

[1] Kar A.K. (2016). Bio inspired computing—A review of algorithms and scope of applications. Expert Syst. Appl..

[2] Figueroa-Mata G., Mata-Montero E. (2020). Using a convolutional siamese network for image-based plant species identification with small datasets. Biomimetics.

[3] Gao W., Xu C., Li G., Zhang Y., Bai N., Li M. (2022). Cervical Cell Image Classification-Based Knowledge Distillation. Biomimetics.

[4] Khan A.T., Cao X., Liao B., Francis A. (2022). Bio-Inspired Machine Learning for Distributed Confidential Multi-Portfolio Selection Problem. Biomimetics.

[5] Coto-Jiménez M. (2019). Improving post-filtering of artificial speech using pre-trained LSTM neural networks. Biomimetics.

[6] Harb A., Fakhreddine M., Zaraket H., Saleh F.A. (2022). Three-dimensional cell culture models to study respiratory virus infections including COVID-19. Biomimetics.

[7] Bulut A., Temur B.Z., Kirimli C.E., Gok O., Balcioglu B.K., Ozturk H.U., Uyar N.Y., Kanlidere Z., Kocagoz T., Can O. (2023). A Novel Peptide-Based Detection of SARS-CoV-2 Antibodies. Biomimetics.

[8] Influenza on the Rise in Multiple Regions Across China, Dominated by H1N1 Virus. https://www.globaltimes.cn/page/202302/1286396.shtml.

[9] Becker N.G., Watson L.F., Carlin J.B. (1991). A method of non-parametric back-projection and its application to AIDS data. Stat. Med..

[10] Kim S., Byun J.H., Jung I.H. (2019). Global stability of an SEIR epidemic model where empirical distribution of incubation period is approximated by Coxian distribution. Adv. Differ. Equ..

[11] Sun H., Qiu Y., Yan H., Huang Y., Zhu Y., Chen S. (2020). Tracking and predicting COVID-19 epidemic in China mainland. MedRxiv.

[12] Chen Y., Lu P., Chang C., Liu T. (2020). A Time-Dependent SIR Model for COVID-19 With Undetectable Infected Persons. IEEE Trans. Netw. Sci. Eng..

[13] Giordano G., Blanchini F., Bruno R., Colaneri P., Di Filippo A., Di Matteo A., Colaneri M. (2020). Modelling the COVID-19 epidemic and implementation of population-wide interventions in Italy. Nat. Med..

[14] Becker N.G., Marschner I.C. (1993). A method for estimating the age-specific relative risk of HIV infection from AIDS incidence data. Biometrika.

[15] Chau P.H., Yip P.S., Cui J.S. (2003). Reconstructing the incidence of human immunodeficiency virus (HIV) in Hong Kong by using data from HIV positive tests and diagnoses of acquired immune deficiency syndrome. J. R. Stat. Soc. Ser. (Applied Stat.).

[16] McEwan P., Ward T., Chen C.J., Lee M.H., Yang H.I., Kim R., L’Italien G., Yuan Y. (2014). Estimating the incidence and prevalence of chronic hepatitis C infection in Taiwan using back projection. Value Health Reg..

[17] Chau P.H., Li W.Y., Yip P.S. (2020). Construction of the infection curve of local cases of COVID-19 in hong kong using back-projection. Int. J. Environ. Res. Public Health.

[18] Marschner I.C. (2020). Back-projection of COVID-19 diagnosis counts to assess infection incidence and control measures: Analysis of Australian data. Epidemiol. Infect..

[19] Shahid F., Zameer A., Muneeb M. (2020). Predictions for COVID-19 with deep learning models of LSTM, GRU and Bi-LSTM. Chaos Solitons Fractals.

[20] Kapoor A., Ben X., Liu L., Perozzi B., Barnes M., Blais M., O’Banion S. (2020). Examining COVID-19 forecasting using spatio-temporal graph neural networks. arXiv.

[21] Gao J., Sharma R., Qian C., Glass L.M., Spaeder J., Romberg J., Sun J., Xiao C. (2021). STAN: Spatio-temporal attention network for pandemic prediction using real-world evidence. J. Am. Med. Inform. Assoc..

[22] Panagopoulos G., Nikolentzos G., Vazirgiannis M. Transfer graph neural networks for pandemic forecasting. Proceedings of the AAAI Conference on Artificial Intelligence.

[23] Fu B., Yang Y., Ma Y., Hao J., Chen S., Liu S., Li T., Liao Z., Zhu X. Attention-based recurrent multi-channel neural network for influenza epidemic prediction. Proceedings of the 2018 IEEE International Conference on Bioinformatics and Biomedicine (BIBM).

[24] Deng S., Wang S., Rangwala H., Wang L., Ning Y. (2019). Graph message passing with cross-location attentions for long-term ILI prediction. arXiv.

[25] Tian T., Jiang Y., Zhang Y., Li Z., Wang X., Zhang H. (2020). COVID-Net: A deep learning based and interpretable predication model for the county-wise trajectories of COVID-19 in the United States. MedRxiv.

[26] Wu Y., Yang Y., Nishiura H., Saitoh M. Deep learning for epidemiological predictions. Proceedings of the 41st International ACM SIGIR Conference on Research and Development in Information Retrieval.

[27] Zheng N., Du S., Wang J., Zhang H., Cui W., Kang Z., Yang T., Lou B., Chi Y., Long H. (2020). Predicting COVID-19 in China Using Hybrid AI Model. IEEE Trans. Cybern..

[28] Gatta V.L., Moscato V., Postiglione M., Sperli G. (2021). An epidemiological neural network exploiting dynamic graph structured data applied to the COVID-19 outbreak. IEEE Trans. Big Data.

